# In the eye of the beholder: to make global health estimates useful, make them more socially robust

**DOI:** 10.3402/gha.v9.32298

**Published:** 2017-05-22

**Authors:** Elizabeth Pisani, Maarten Kok

**Affiliations:** a The Policy Institute, King’s College London; b Institute for Health Policy and Management, Erasmus University Rotterdam; c Talma Institute, Vrije Universiteit Amsterdam

**Keywords:** Bringing the indicators home: Country perspective on the utility of global 40 estimates for health indicators (WHO), Political economy, world health organization, monitoring and evaluation, sustainable development goals

## Abstract

A plethora of new development goals and funding institutions have greatly increased the demand for internationally comparable health estimates in recent years, and have brought important new players into the field of health estimate production. These changes have rekindled debates about the validity and legitimacy of global health estimates. This paper draws on country case studies and personal experience to support our opinion that the production and use of estimates are deeply embedded in specific social, economic, political and ideational contexts, which differ at different levels of the global health architecture.

Broadly, most global health estimates tend to be made far from the local contexts in which the data upon which they are based are collected, and where the results of estimation processes must ultimately be used if they are to make a difference to the health of individuals. Internationally standardised indicators are necessary, but they are no substitute for data that meet local needs, and that fit with local ideas of what is credible and useful. In other words, data that are both technically and socially robust for those who make key decisions about health.

We suggest that greater engagement of local actors (and local data) in the formulation, communication and interpretation of health estimates would increase the likelihood that these data will be used by those most able to translate them into health gains for the longer term. Besides strengthening national information systems, this requires ongoing interaction, building trust and establishing a communicative infrastructure. Local capacities to use knowledge to improve health must be supported.

## Background

The validity and legitimacy of global health estimates have been a topic of debate for at least two decades [1–5], but it was the Global Burden of Disease estimates of 2010 that really set the discussions alight. The publication by the Institute for Health Metrics and Evaluation (IHME) of estimates for the burden of very many diseases in very many countries drew sharp responses, in two waves. The first wave focused largely on technical issues. Academics and health officials from several countries were confronted with estimates they found hard to reconcile with the facts as they saw them; this led to many questions about data sources [6–10]. Experts working globally on specific disease areas questioned methods, complaining that they could not see the workings inside the ‘black box’ of IHME models [3,11]. Rumbling under both of these areas of concern was a larger discomfort, which built into a second wave of responses, questioning power relationships in global health.

The second wave of responses focused mostly on social issues, such as the role of health estimates in shaping the global health agenda. Who is making the estimates, and by what right? How ‘robust’ are they, and how ‘legitimate’ [12–20]? Several contributors to this debate recognised that data and concepts in global health are institutionally and politically constructed: a health issue rises up the international agenda because people deemed to be experts have used accepted methods to demonstrate its importance, and have communicated that in forums which entrench that importance (and which influence funding decisions). But there has been less discussion of how these constructions come about. Who designates the experts? Which methods are considered robust? Which forums confer legitimacy to communicated data? Whose funding decisions are influenced?

A few authors have argued that the political legitimacy and technical validity of global health estimates would be improved if estimation processes worked from the bottom up [2,11,20]. However most of the debate so far has centred on the interests of institutions and individuals who work at a supranational level, as though ‘global health’ were in some way independent of the health of billions of individuals living in specific local and national settings, as though global health estimates were independent of data collected by people and institutions in very concrete contexts. We believe that health data and estimates at any level are only useful if they are demonstrably used to improve the health of individuals other than those (including ourselves) who make a comfortable living out of the health estimates industry. We thus turn our attention to what makes health estimates useable, and useful.

In this opinion paper, we draw on the country case studies presented in this volume, on our own work in countries as diverse as China, Indonesia and Ghana, and on discussions with health officials from middle-income countries and international organisations described at greater length in this volume by Abou-Zahr and Boerma, to examine which health data prompt changes that lead to better health [21]. (Phrases in italics are verbatim quotes from discussants.) We argue that health estimates are deeply embedded in specific social, economic, political and ideational contexts that differ at different levels of the global health architecture [22]. What is considered legitimate, robust and useful thus differs also. We introduce the concept of ‘social robustness’ and suggest ways in which these different norms might be aligned so that the needs of different actors can be met without undermining one another.

## What makes estimates ‘robust’? Commonalities across contexts

Health data, including estimates, are produced by a variety of organisations whose mandates, aims, incentive structures and institutional cultures differ. These differences shape both the processes through which data are collected and analysed, and the interpretation of the results. Health data are often presented as ‘objective’, but like all other knowledge they are a construct that derives meaning from the very process of its construction.

This process of construction, the interpretation of data and their perceived utility are shaped by the actors involved, their priorities and the institutions and circumstances in which they are embedded. Institutional priorities are themselves shaped by similar (often interacting) factors. The most salient questions to ask in understanding how and why priorities relating to the production of knowledge may differ include the following.

### Who chooses the questions, and what’s their goal?

Data are produced in response to some perceived need, which must be articulated in questions that determine what data are collected, and in analyses determining how they will be understood. Those choosing the questions may or may not be the end users of the data; but their interests and aims will certainly influence the utility of the data to all potential users.

### Who pays for the data collection and knowledge production?

The source of funding often (though not always) strongly influences the questions that get asked, and the ways in which they get answered [23,24]. Health authorities may need to take into account the interests and concerns of tax-payers, politicians or external funding agencies in planning knowledge production; these interests can lead to the overemphasis or neglect of different types of information, health issues and populations.

### Who produces the data/knowledge?

Data and knowledge producers are driven by a variety of personal, professional and institutional incentives: the reward system for academics has little in common with that of national health authorities. These differences can affect the timing as well as the nature of knowledge production.

### How are the data communicated, by whom, to whom?

Communication is an inherent part of the process of knowledge production; it confers meaning on raw information. The perceived credibility of health data is very much influenced by the format of its communication, the communicator, and the interaction between the communicator and the audiences, each of which will understand the data within the framework of their existing worldview.

### How are the data used, by whom and for whom?

The same data can acquire meaning and utility in various ways that are not always consistent with the aims of their producers.

Though the constellation of actors and factors involved in producing data is by definition locally specific and deeply contextual, it does tend to manifest in broadly ‘typical’ patterns for different data producers at different levels of the global health architecture. Next, we examine three typical constellations and suggest how they affect the perceived legitimacy of data outputs, and their utility.

## Sub-national and national levels

At country level, the most important function of health data is to inform the prospective planning and continual evaluation and adjustment of health service provision. While questions may be determined at the national level, data are most commonly used at the sub-national level: ‘For us, the national is nothing’ (health official, Latin America).

In middle- and higher-income countries, sub-national data collection is part of the routine function of health systems funded out of routine government spending. In low-income countries they may be externally funded through international survey programmes such as the Demographic and Health Surveys (DHS) or the Multi-Indicator Cluster Surveys. These surveys, like routine data collection, are generally carried out by government staff. This creates an institutional imperative to use the data: they are locally owned, produced by colleagues who may be directly involved in communicating results and who can help explain anomalies in the data and their meaning in the specific local situation [25]. These empirical data are seen as robust in that they are concrete, easy to interpret and directly relevant to the local context [26]. However, survey data are rarely representative at the district level at which decisions about service provision are increasingly being made.

The idea of the ‘concrete’ affects the communication of sub-national and national data. Since they are often empirical measures or simple adjusted measures, sub-national and national health data are commonly presented ‘as is’, with limited recognition of the uncertainty associated with the measures. Complex modelled estimates of the type produced by international agencies do include measures of uncertainty, but they are seen by national policy-makers as the product of smoke and mirrors, and mistrusted. ‘If you’ve just done a DHS, you don’t really want to hear about an estimate’ (health official, Africa). They are also seen by national authorities as complicating the communication of health data: ‘I can’t say in parliament or to the media that the indicator is either 40% or 100%. That implies that we don’t know. It’s just not possible. We pick a number and that’s it. We’re certain’ (health official, Latin America).

The media, for their part, are not always convinced by this certainty. They understand that in-country data producers are themselves embedded in a political system, and are often under strong pressure to report statistics that support the political powers of the day. ‘If the national policy is to expand ARV [antiretroviral] coverage, well, it is hard to interpret [the data] against that’ (health official, Asia).

National interest can also affect the likelihood that data will be communicated at all, especially in the case of infectious disease outbreaks. Severe acute respiratory syndrome, avian influenza, cholera and Ebola all provide examples in which countries were initially reluctant to share health data with global health authorities because they feared the economic, social and political consequences that can come with containment measures.

Of all the types of knowledge produced, locally determined empirical measures are most likely to be used in ways that directly affect health service provision. They can sometimes be aggregated upwards to meet national and international needs, although local specificities do not always map neatly onto the standardised, comparable measures required at other levels of the global health architecture.

## International organisations

United Nations (UN) organisations, and especially the World Health Organization (WHO), are mandated by their member states to track the state of health internationally. The information they generate falls into two broad categories: occasional ‘*tours d’horizon*’ of issues of current interest, and regular reporting on key indicators. The appetite for both has grown enormously in recent years, in the former case because voluntary bilateral and philanthropic donors now provide over three quarters of the institution’s annual budget, and make demands of it according to their particular interests. One year, national health ministries will be bombarded with requests for data about drowning. Another year, the demand may be for data about dental or mental health [27–29]. We are not aware of any rigorous evaluations of the costs of these focused estimation exercises, nor of their policy outcomes at the national level.

The regular reporting of internationally agreed indicators such as the Sustainable Development Goals has become the bread and butter of statisticians at the WHO and other specialist UN agencies. There is a huge demand for these estimates, which involve the regular publication of standardised indicators that allow all UN member states to be compared at a single point in time. Organisations that fund development efforts demand indicators with a regularity incompatible with lengthy consultation. They also want a level of comparability not easily achieved given the diversity (and sometimes the sheer absence) of underlying data. The estimation process is fiercely political, because the results of these estimates are used to ‘grade’ country progress towards agreed goals, and to rank the relative importance of conditions in which money will be invested and in which different institutions – including within the UN ‘family’ – have an interest.

The types of data produced by the WHO and some other specialist UN organisations are greatly influenced by a mandate that goes beyond the simple compilation of country-reported statistics: these organisations seek to add value through technical advice. This institutional culture has led to a huge investment of time and energy in developing health estimates that are technically robust. Together with the imperative to publish comparable statistics on a very regular basis, this focus on the technical has undermined consultation and other social processes which might increase the utility and uptake of data at the national level.

Currently, international organisations most commonly communicate data in published reports that are positioned for maximum public exposure, including through the media. The media, which see the WHO and other UN organisations as the repositories of technical expertise, generally oblige. National authorities, however, are sometimes blind-sided by internationally comparable estimates, which often derive from a country consultation that is little more than cursory, and that differ from the figures used locally. National authorities are sometimes unable to respond appropriately because they do not understand the ‘black box’ which produced the data. The WHO and other producers of health estimates are actually rather transparent about their methods, making them available on websites and sometimes publishing them in the scientific literature. However these publications are usually highly technical, often only in English, and rarely give details of country-level adjustments. A health official from Europe expressed frustration at international organisations’ failure to consult with or explain their methods to national experts:

They ask for data from us, and then they publish without even showing us first, without a chance to give feedback… We were told there was no new data [used in the estimates], only the quality assessment had changed. But there were no methodology notes that told us how or why.

Internationally standardised health data (including those produced by standardised surveys and academic institutions, to be described later) are most useful to development agencies including large private foundations such as the Bill and Melinda Gates Foundation, multi-funder organisations such as Global Fund for AIDS, TB and Malaria and bilateral agencies such as the United States Agency for International Development. Admirably, these institutions want to identify areas of need and to compare different investment opportunities, as well as to track the health impact of initiatives they support. Indeed the desire to increase accountability and show measurable results has been a major driver of the huge rise in demand for these sorts of data; some global health funds use these estimates not just to guide the allocation of resources but to withdraw funding if countries don’t meet numerical targets set and measured through internationally produced estimates.

National governments also use externally made estimates as stop-gaps for areas which have been relatively neglected by local health information systems, and as an advocacy tool: ‘Global estimates are completely useless for planning, but they are useful for political lobbying’ (health official, Latin America). Benchmarking national progress using global comparisons can help secure continued support from national authorities for successful programmes.

## Academic institutions

Academic institutions have long collaborated with national health authorities in generating health-related data and knowledge, and international organisations have also sought advice from academics in developing methods and estimates. In these instances, academics answer questions posed by their partners. Many also develop research agendas driven by their personal interests.

More recently, specialised global health research institutions situated within academic institutions have become important producers of health estimates and statistics, often in collaboration with UN and other multi-country organisations. Examples include the London School of Hygiene and Tropical Medicine, the Johns Hopkins Bloomberg School of Public Health and the Norwegian Institute of Public Health. The most significant recent arrival is the IHME, based at the University of Washington in Seattle. IHME is funded primarily by the Bill and Melinda Gates Foundation to the tune of some US$ 35 million a year; the main thrust of its work to date has been to produce the standardised and internationally comparable data that the private foundation needs to inform its investment choices.

IHME is staffed by hundreds of well-trained data scientists and analysts who have access to teraflops of computer processing power; it is thus supremely well placed to develop new data processing and statistical modelling methods. Though feathers have been ruffled, especially among the international organisations that had enjoyed a near monopoly on technical expertise at the international level since global estimates became fashionable in the 1990s, IHME has done a great deal to push forward the technical frontiers of health estimates production. To this extent, their estimates compete with those of the WHO and other international organisations to be considered the most technically robust. Data produced by IHME and other academic institutions have in some cases forced actors in both governments and international organisations to revisit their own data and methods, and to make both more transparent.

Academic researchers are to a great extent driven by the incentives of their profession, which reward publishing papers in peer-reviewed journals, regardless of whether or not the data are used to improve health outcomes. This necessarily influences the communication of results. High-level, multi-country comparisons have proven attractive to the editors of high-profile journals, *The Lancet* in particular [30–36]. Publication in these journals in turn imbues academic estimates with a legitimacy that is not shared by data produced at the country level.

When academic analyses are demand driven, for example when national governments outsource their data collection and analysis to academic institutions or where there is a genuine collaboration between academic institutions and end users, the results are likely to be valued and used to influence a country’s policy choices. In Ghana, for example, a detailed impact assessment of 30 studies showed that research was most likely to be used when it was aligned to national priorities and led by people embedded in the contexts in which results can be used [37].

Collaboration between academic, government and civil society partners can also increase the credibility of estimates in the public view, because citizens perceive academics to be more objective than civil servants, while the inclusion of non-government voices keeps the process ‘honest’. In Indonesia, for example, HIV estimates made in Geneva at the start of this decade were largely ignored by a government uninvolved in their production. A national estimation process led by the Ministry of Health but involving academics, non-government organisations, the police, the narcotics control board and others led to technically sound estimates that were widely accepted because so many deeply disparate groups had contributed data, argued over methods and agreed on the outcome [38].

Increasingly, IHME is working with governments to produce health estimates at the sub-national level – China, Mexico and the United Kingdom are recent examples. These are all countries with relatively strong health information systems; they have the data to produce meaningful local estimates, as well as the indigenous capacity to interpret and use model outputs. If countries such as these can learn from academic organisations the skills needed to run sophisticated models, the increased understanding of national diversity that results will doubtless be illuminating, and perhaps even be useful and used.

The same is not true when these models are used by countries with poor data. Standardised models that use estimated parameters to produce comparable data for close to 200 countries inevitably iron out precisely the differences and nuances that are most important for local decision-making. Trying to correct for that by making sub-national estimates in data-poor settings simply multiplies the likely inaccuracies.

## Utility, in the eye of the beholder

**Figure 1.**
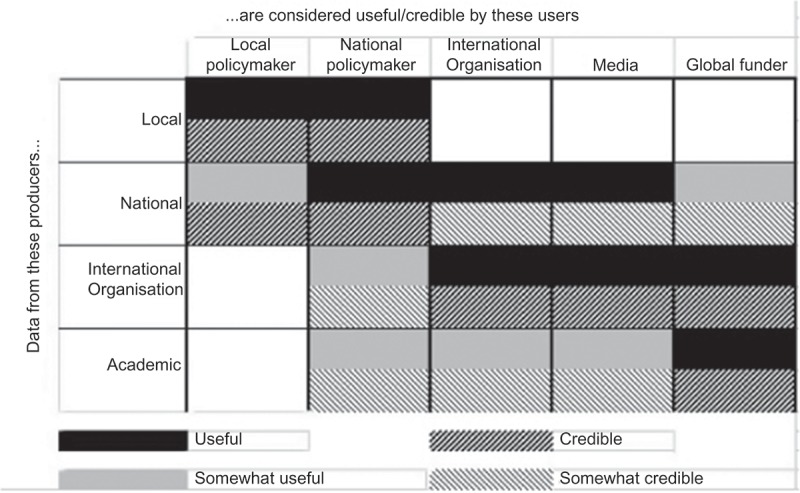
Perceived utility and credibility of health data and estimates to various users, by institutional level at which data are produced.

From the foregoing discussion, we see that health data that are considered high quality and valid by some, are considered more or less useless by others; we have found this in our own experience, but it is reflected also in the literature and in the widely differing opinions that were expressed even within the discussion which led to this paper. Figure 1 tries to summarise graphically these opinions about how different potential users see health estimates made by knowledge producers at various levels. We make a distinction between credibility – the belief that an estimate provides a good approximation of reality – and utility – the potential for the data to be acted upon in ways that may contribute to improved health.

A traditional and technocratic view would hold that data must be credible to be useful, and, further, that if data do reflect reality, they are likely to be useful. Decades of studies of the use of research have shown that the relationship is not so simple [22]. Consider the example of a journalist who is aware that an estimate in an academic report is the product of guesstimated data adjusted using an assumed parameter. They may not find that estimate particularly credible, but it may be useful to them because it provides comparability and comes with seductive visualisations. Another example: consider the case of local health officials who know that estimates made by academics are far more accurate than their own official reports, but who are unable to act on the ‘credible’ data because of local political constraints (a situation which arose, for example, during the outbreak of HIV among plasma donors in central China [39]).

The extent to which data are believed and used depends not just on their technical quality. It depends also on whether and how data are communicated to users; on whether and how those users trust the source of, understand and accept the data; and on the extent to which they have the capacity to combine data with existing knowledge and give meaning to them for their specific aims in their specific local situation [37,40]. These processes are in turn shaped on the demand side by the political and institutional cultures of potential users and their perceived need for the data. On the supply side, they are shaped by the constellation that is calling the shots intellectually, financially and institutionally.

In short, the moment we become interested in the actual use of data and their contribution to action, we are forced to look beyond the technical and take into account the social robustness of data. Technical robustness is necessary, but it is not sufficient and does not exist in a vacuum; without people who are aware of, understand and accept data, they have no meaning. When we take into account the socio-political dimensions of knowledge, we are obliged to confront the fact that narrowly technical processes produce data that are only valued by very specific groups in very specific situations.

## Towards socially robust data

The perceived robustness of health data is, as we have seen, a movable feast: different actors come to the table with different needs, expectations and professional norms, both on the supply side and on the demand side. In principle, there is no harm in the fact that various producers are generating data to meet the need of different consumers. Private organisations have every right to spend money supporting the analysis they believe they need to guide investment decisions. International organisations are bound to fulfil their mandates to provide member states with comparable indicators of progress towards internationally agreed goals. Countries inevitably invest public money in generating the granular information they need to plan and deliver health services locally.

The problem arises when these processes duplicate or, worse still, undermine one another; at the moment, both are happening. Duplication occurs in part because international organisations and academic groups driven by a seemingly implacable need for comparable data are unwilling to accept from countries contributions which do not meet global definitions or standards. Using imputation and other statistical techniques, they substitute estimates made in Geneva or Seattle for those made in Ankara or Pretoria. One must assume that the groups publishing these substitute estimates consider them to be of better quality than the originals, perhaps because they have been technically validated. But substitute estimates are often made with minimal consultation with the people who provided the data which were fed into the maw of the multi-country model – people who could point out specificities, biases or alternative data sources that should be considered in interpreting the validity of the modelled outputs. Academic researchers from one African country gave an example: an international group estimated mortality at a figure lower than the number of deaths actually registered by the country’s incomplete vital registration system. Local researchers suggested adjustments to data inputs based on their own granular knowledge of what is and is not captured in national health statistics. ‘But we hit the roadblock of one size fits all. We were told: “we can’t change things just for [your country], it has to fit into this big global model”’ (academic researcher, Africa).

Neither engaged in the process of making new estimates nor fully understanding the complexity of the model, people responsible for health data at the local level have little incentive to push for action based on the outcome. Where the substitute estimate is better than the original, this represents a wasted opportunity for learning and improvement at the country level. But as long as countries have locally driven data to fall back on, the existence of substitute estimates is not fatal.

In many cases, however, estimates exist because reliable measured data don’t. This is in part because of decades of under-investment in local health information systems, including by the very organisations that now demand annual estimates. ‘Of the 40 or so countries that account for 90% of child mortality, only a handful have functioning vital statistics systems,’ noted a senior official from one international organisation. ‘It’s borderline criminal for donors to be bragging about anything having to do with data when their billions have left that kind of legacy.’ In weaker countries, running complex computer programmes to generate ‘data’ intended to fill information gaps can form part of a vicious circle which leads to ever weaker health information. If under-resourced ministries of health, planning or finance see apparently credible groups using computing power to make up estimates in the absence of measured data, they are unlikely to invest in health information systems. And without local engagement in data collection and interpretation, there’s little chance that data will be understood and integrated into local systems of belief and action. It’s worth noting that engaging data collectors in an active feedback loop probably increases the technical validity of the data also: nurses and other frontline workers who collect and report the raw information on which estimates should be based are more likely to fulfil that apparently peripheral task diligently if they see the data being used to improve service provision.

It is the process of engagement with different actors, their institutional contexts, political imperatives and belief systems, that makes data socially robust.

### 

**Box 1.** The pillars of socially robust knowledge

**Figure 2.**
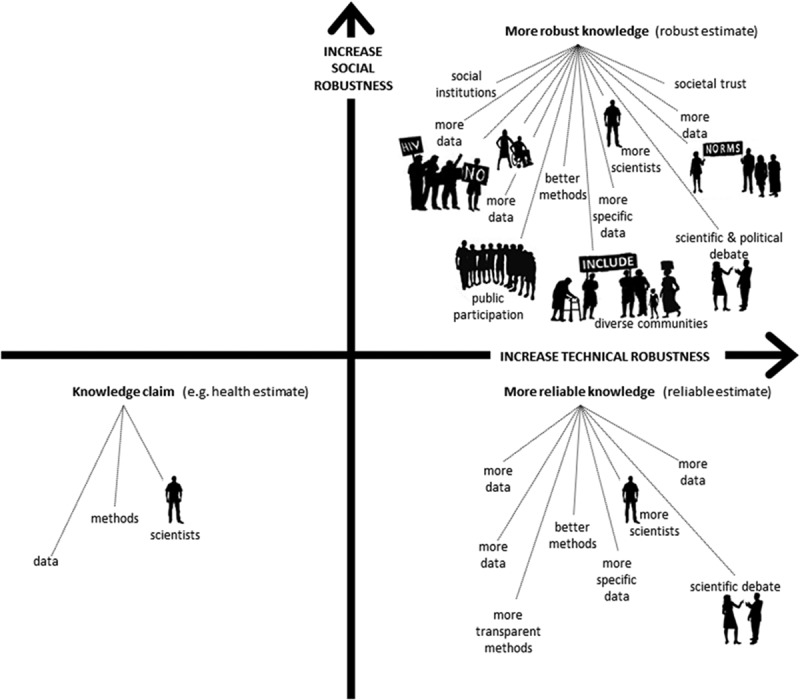
A graphic representation of the robustness of knowledge.

The ‘robustness’ of a knowledge claim is similar to that of other constructions, such as a bridge. The more well-constructed pillars there are supporting a bridge, the more likely it is to be robust. Our confidence in the construction is increased after the bridge has been tested by a variety of vehicles in different weather conditions.
Scientific knowledge is also constructed: the solidity of scientific achievements is a matter of alignment between data, arguments, interests, dominant values and circumstances [41]. The quality and validity of knowledge are made, and the ‘robustness’ of such constructions is tested, through ongoing debate, new research and the challenges that arise when the knowledge is acted upon.
Scientists tend to consider a knowledge claim more robust when it is based upon more and increasingly specific data, and constructed using ever-improving technical methods. Scientific standards and norms are not always universally agreed even within the scientific community, however, hence the importance of transparency about methods and data, which allows others to test a knowledge claim.
Once the ‘knowledge’ produced by scientists migrates outside of the research community, it faces a broader challenge: it must link up to what matters for those people who make decisions about health policy and practice in concrete local circumstances. In other words, it will be tested against social as well as scientific standards. If those standards have been taken into account when designing the pillars that underpin the new construction, that knowledge will function better in the real world.
Knowledge is always linked to concrete practices and institutions, and has to be understood, accepted and trusted by real people in the broader context of their daily lives and beliefs. As Figure 2 illustrates, knowledge becomes more socially robust when more people, from more diverse communities and institutions with a wider variety of worldviews and practices, understand, accept and trust it, and find it useful for their own aims in their own situation.

## Aligning interests

In discussions around the utility and legitimacy of health estimates, ‘inclusiveness’ is sometimes set in opposition to ‘productivity’. In other words, the sort of engagement that produces socially robust estimates (inclusiveness) can undermine the timely production of technically accurate estimates (productivity). We contend that the two are not opposed, because we believe there is nothing productive about pumping out technically credible data that are not used to improve health. Social and political engagement are not substitutes for technical validity, but are an integral component of data and knowledge that are widely accepted and used.

The balance of emphasis may vary by setting, because the different institutional imperatives of the various data producers and users are not going to disappear. However, we would argue that more inclusive engagement could turn a vicious into a virtuous circle, beginning with greater investment in local data production, interpretation and use.

At the local and national levels, data producers must first and foremost meet the data needs of the policy-makers who decide how resources will be allocated between local communities and health priorities. To do that well, they must by definition engage with both the policy-makers and the local communities; effective local knowledge-generation processes are thus inherently the most socially robust among those who make key decisions. Much locally produced data can be aggregated upwards to meet national and international needs. This is not always the case, however. (Brazil, for example, reports the percentage of women who have had seven antenatal visits rather than the globally standardised indicator based on four visits.) In this case engaging international actors can lead either to acceptance by supra-national bodies of local standards or to technical and/or financial support for new data collection efforts, where these would represent a benefit to local actors also.

At the international level, meaningful engagement may slow the process, but it will improve the result. The WHO and other international organisations are wary of prolonged consultation in part because it disrupts the production schedule demanded by their paymasters, and in part because ministries of health often pressure them to publish estimates deemed politically acceptable. We believe that’s an argument for more consultation, not less. The widest possible engagement, including with academic institutions and civil society organisations at the national level, protects against lopsided political pressure. It also makes use of local knowledge; that’s generally an improvement on the knowledge generated by a globally standardised computer programme. The experience in developing national and sub-national HIV estimates, described by Mahy and colleagues in this volume, provides strong evidence of the effectiveness of this approach.

Achieving these changes would require the institutions that demand internationally comparable data from international organisations – mostly UN member states and their development organisations but also private foundations and multilateral health funds – to recognise that socially robust processes may result in slightly lower frequency and even somewhat less standardised measures, even as they lead to more use of data to guide service provision locally and nationally. We note that many richer nations do not themselves report health data in the formats required of most low- and middle-income nations, so they should have little difficulty understanding the utility of local variation.

It is unclear how we should go about developing socially robust processes in places where there are no data at all, including in areas of conflict and failed states. At the moment in these situations, we simply make up figures with the help of computer models. This does not seem useful: where there are no data, there is unlikely to be the capacity or the will to act on data produced with no reference to whatever limited social or political structures may be in place. If the international community is not willing or able to work with local powers to develop health information systems, then it should perhaps be willing to live with blanks in its global disease estimates.

Systematic monitoring of the use of health data, and further case studies of how information systems at different levels co-evolve and can be strengthened, may help to guide efforts and investments, stimulate a virtuous cycle and enhance the likelihood that research contributes to action. Existing methods for monitoring the use of research have been used in both high- and low-income countries [37,42,43].

In summary, if we wish health estimates to be used to improve health, then it is not enough to publish technically robust indicators in journals and the reports of international organisations. Estimates must be arrived at through a process that is understandable and relevant to the people who can act on the data to improve health policies and programmes. This requires ongoing interaction, trust and a communicative infrastructure that support genuine consultation and dialogue, not just between producers of global estimates and national health authorities, but within countries, between those who have knowledge to contribute – very often groups with a stake in the outcome, who are also in a position to promote appropriate action.

## Paper context

This paper arose from a meeting convened by the World Health Organization and entitled “The utility of estimates for health monitoring and decision-making: global, regional and country perspectives.“ The meeting, held in June 2015, brought health officials from low- and middle-income countries together with technical staff from WHO, IHME and other organisations that make health estimates at the global level. Both authors attended the meeting and were struck by the very different ways in which participants representing national and global organisations perceived the utility of health estimates.
